# Echinacoside Alleviates Metabolic Dysfunction-Associated Steatotic Liver Disease by Inhibiting Ferroptosis via Nrf2/HMOX1 Pathway

**DOI:** 10.3390/biomedicines12122728

**Published:** 2024-11-28

**Authors:** Yiming Yan, Ningxi Yang, Fanglin Qin, Yarong Hao

**Affiliations:** Department of Geriatric, Renmin Hospital of Wuhan University, No. 99 Zhangzhidong Road, Wuchang District, Wuhan 430060, China; yanyiming98@126.com (Y.Y.); cxlgohigher@163.com (N.Y.); fanglinq0723@163.com (F.Q.)

**Keywords:** metabolic dysfunction-associated steatotic liver disease, echinacoside, ferroptosis, mitochondria, Nrf2, GPX4

## Abstract

**Background:** Metabolic dysfunction-associated steatotic liver disease (MASLD) is a chronic liver disease characterized by hepatic lipid accumulation, and echinacoside (ECH) has demonstrated antioxidant and anti-inflammatory effects across multiple conditions, it has demonstrated hepatoprotective effects. Ferroptosis represents a novel mechanism of cell demise, differing from apoptosis and autophagy. Emerging research indicates that ferroptosis in hepatocytes plays a role in the development of alcoholic liver disease. This study aimed to reveal the effect and potential mechanism of ECH on MASLD. **Methods:** The effect of ECH on the viability, lipid deposition, lipid peroxidation, mitochondrial of OA/PA-treated HepG2 cells were evaluated by Cell Counting Kit-8 assay, JC-1 and immunofluorescence assay. Meanwhile, the mechanism of ECH was assessed using transmission electron microscopy and immunofluorescence analysis. Moreover, db/db mice, a spontaneous type 2 diabetes mode, were intragastrically administered ECH by 300 mg/kg or an equivalent volume of saline. Body weight, lipids, and liver function were measured. liver pathology was performed. The mechanism of ECH in vivo was analyzed using Western blot and immunofluorescence analysis in db/db mice. **Results:** ECH attenuated lipid deposition, lipid peroxidation and ferroptosis induced by OA/PA in HepG2 cells. Mitochondrial morphology and function in HepG2 cells were also preserved by ECH. In db/db mice model of MASLD, ECH markedly ameliorated liver hepatocellular ballooning, inflammatory cell infiltration in the portal area, and fibrous tissue proliferation. ECH also increased the expression of Nrf2, HMOX-1, SLC7A11, and GPX4, and decreased the expression of ACSL4 in liver tissues. Mechanically, ECH repressed ferroptosis by activating the Nrf2/HO-1 signaling pathway. **Conclusions:** Our research revealed that ECH has the capability to modulate ferroptosis via the Nrf2-HMOX1pathway, consequently mitigating the progression of MASLD. This suggests that ECH has a potential role in the treatment of MASLD.

## 1. Introduction

Metabolic dysfunction-associated steatotic liver disease (MASLD) comprises a spectrum of liver disorders associated with metabolic syndrome, presenting a growing disease burden nationally and globally. Its main pathologic manifestation is hepatic steatosis, which is a common, nonspecific lesion. As the disease progresses, metabolic dysfunction-associated steatohepatitis (MASH), characterized by liver inflammation, hepatocellular injury, and fibrosis, may also develop, which can advance to hepatic fibrosis, cirrhosis, and even hepatocellular carcinoma (HCC), underscoring its potentially progressive nature. Histologic features of MASLD include macrovesicular steatosis, hepatocellular ballooning, scattered inflammation (mainly lobular), apoptotic vesicles, Mallory–Denk bodies (MDB), and even tissue fibrosis [1]. The pathogenesis of MASLD is very complex, and there have been many theories, but the “second strike theory” is the most recognized pathogenesis. The first blow is that fatty acids (FA) are deposited as triglycerides (TG) in the liver parenchymal cells after entering the liver, and intracellular lipid metabolism is disturbed. When cells are unable to store large amounts of free fatty acids in the form of TG or exceed the cellular oxidative load, excess fatty acids and large amounts of reactive oxygen species (ROS) cause cellular lipid peroxidation, oxidative stress, endoplasmic reticulum stress, ferroptosis, and apoptosis, which is the second blow to the development of the disease [2]. Disturbed hepatic lipid metabolism and further triggering a series of abnormal consequences are the key factors in the development of MASLD, so the ferroptosis of hepatic cells is closely related to the development of MASLD.

Ferroptosis represents an iron-dependent programmed cell demise, distinct from conventional cell death modalities such as apoptosis, thermoapoptosis, autophagy, senescence, and necrosis [3,4]. Intracellular iron overload triggers the Fenton reaction to generate excess lipid peroxides, and the iron chelator deferoxamine inhibits cell death due to iron-dependent ROS and lipid ROS accumulation [5]. Numerous iron metabolic processes, including iron acquisition, utilization, storage, and expulsion, impact the equilibrium of the intracellular iron reservoir, thereby influencing cellular susceptibility to ferroptosis. Iron acts as a cofactor that is critical for the function of certain essential enzymes, such as the iron-containing enzymes lipoxygenase (ALOX), EGLN prolyl hydroxylase (PHD), and cytochrome P450 oxidoreductase (POR) [6]. ALOXs act as oxygenases critical for initiating iron metabolism by oxidizing PUFA-PE (polyunsaturated fatty acid-phosphatidylethanolamine), thereby initiating the ferroptosis cascade, a key process in ferroptosis. In addition, the synthesis of PUFA-PE depends on the activation of ACSL4 [7]. Remarkably, among saturated fatty acids, monounsaturated fatty acids (MUFAs), and polyunsaturated fatty acids (PUFAs), PUFAs emerge as the primary targets of lipid metabolism in the iron-mediated metabolic response [8]. The cysteine/glutamate transporter system xc- consists of a heavy chain (4F2, gene name SLC3A2) and a light chain (xCT, gene name SLC7A11), which are responsible for the synthesis of the most important endogenous antioxidant, GSH. Identified iron metabolism modulators (Erlastine, Sulfasalazine, and Sorafenib), also proposed as inhibitors of system xc-, impede the expression of SLC7A11, consequently resulting in GSH depletion [9]. Other upstream regulators of SLC7A11 have been identified, including P53, NFE2L2, BAP1, NRF2, HIFs, and YAP1. Targeting SLC7A11 could potentially serve as a treatment strategy for various diseases by inhibiting iron metabolism [10,11]. Glutathione peroxidase 4 (GPX4) is an antioxidant enzyme that requires selenium to function. It plays a crucial role in directly reducing phospholipid hydroperoxides into non-toxic phosphatidyl alcohols within plasma membranes and various intracellular organelle membranes. GPX4 is widely acknowledged as a key downstream regulator of iron metabolism, primarily through its ability to inhibit lipid peroxidation. This inhibition can be influenced by the depletion of glutathione (GSH) or the regulation of selenium levels [12]. Several studies have confirmed that the upregulation of COX2 serves as a reliable biomarker of ferroptosis, although it is not a determinant of iron metabolism [13]. Notably, mitochondria are involved in cysteine deprivation and erastin-induced iron metamorphosis. Mitochondrial morphological changes, including rupture, reduction, or disappearance of mitochondrial cristae, disruption of the outer mitochondrial membrane, compression or shrinkage of mitochondrial volume, and reduced mitochondrial membrane potential (MMP), are consistent with representative features of mitochondria in iron metamorphosis [14].

Cistanche tubiflora is a traditional plant with hepatoprotective, nephroprotective, and intestinal properties. Echinacoside (ECH), the active component of Cistanche tubiflora, exhibits various biological effects, including antioxidant and anti-inflammatory properties. It has demonstrated hepatoprotective effects by mitigating oxidative stress, inflammation, and cell death [15]. One study evaluated the effect of pinealoside on nuclear factor erythroid 2-related factor 2 (Nrf2), which can inhibit oxidative stress and apoptosis by affecting Nrf2. ECH significantly increased the in vivo and in vitro activity of Nrf2 [16]. Meanwhile, it was found that the expression of GPX4, heme oxygenase-1 (HMOX-1) and glutamate-cysteine ligase catalytic subunit (GCLC) also increased with the elevation of Nrf2 levels, and the expression of GPX4 and HMOX-1 could be effectively up-regulated through the Nrf2 pathway, which could increase the tolerance to iron enrichment and inhibit oxidative stress and apoptosis levels [17,18]. In the present study, we demonstrated for the first time that ECH delayed the development of MASLD by inhibiting ferroptosis and protecting mitochondrial function in hepatocytes through increasing the activity of Nrf2. Therefore, ECH has a broad application prospect for the treatment of MASLD.

## 2. Materials and Methods

### 2.1. Cell Culture

The experimental procedures received approval from the Animal Care and Use Committee of the Renmin Hospital of Wuhan University. HepG2 cells were obtained from Wuhan Procell Life Science & Technology Co., Ltd. (Wuhan, China) (No. PC-H2023081407). Cells were cultured in DMEM high-glucose medium (Servicebio, Wuhan, China) supplemented with 1% penicillin-streptomycin (Servicebio, Wuhan, China) and 10% fetal bovine serum (FBS, Servicebio, Wuhan, China) in a humidified environment containing 5% CO_2_ at 37 °C. After seeding and adherence for 24 h, HepG2 cells were treated with OA/PA (Sigma–Aldrich, St. Louis, MO, USA), ECH (No. 190906, China, Shanghai Medical Science Company, Shanghai, China), 5 μM ML385 (MedChemExpress, Princeton, NJ, USA), and 1 μM Fer-1 (Sigma–Aldrich, USA) for a continuous period of 24 h. The solvent for ECH was ddH_2_O, and the solvent for Fer-1 was DMSO (final concentration below 0.1% (*v*/*v*)).

### 2.2. Cell Viability

The cytotoxicity of ECH and OA/PA on HepG2 cells was detected using Cell Counting Kit-8 (CCK8, Servicebio, Wuhan, China). HepG2 cells were inoculated in 96-well plates at a density of 5000 cells/well and treated with ECH (25, 50, 100, 200, 400 μM) and OA/PA (150/75 μM) for 24 h. After removing the medium, 100 μL of pure medium (containing 10% CCK8) was added to each well and incubated for 2 h at 37 °C. The absorbance at 450 nm was measured using a microplate reader (EnVision, PerkinElmer, Waltham, MA, USA).

### 2.3. Reactive Oxygen Species

DHE was diluted 1:1000 in serum-free medium to achieve a final concentration of 10 μM. The cell culture medium was aspirated, and the appropriate volume of diluted DHE was added. Typically, not less than 1 mL of diluted DHE was added to one well of a 6-well plate and the plate, was then incubated in a cell culture incubator at 37 °C for 20 min. Subsequently, the cells were washed three times with serum-free cell culture medium to fully remove any DHE that did not enter the cells. Finally, observation was conducted using a fluorescence microscope.

### 2.4. Mitochondrial Membrane Potential

A study by Perelman et al. in 2012 found that JC-1 is an ideal and widely used fluorescent probe for detecting mitochondrial membrane potential (ΔΨm). For one well of a 6-well plate, the medium was aspirated, and the cells were washed once with PBS. 1 mL of JC-1 staining working solution was then added with 1 mL of cell culture medium. The mixture was gently mixed. The cells were incubated for 20 min at 37 °C in a cell culture incubator. After incubation, the supernatant was aspirated. The cells were washed twice with JC-1 staining buffer. Finally, 2 mL of medium was added, and the cells were observed under a fluorescence microscope (Evos FL Auto, Waltham, MA, USA).

### 2.5. Transmission Electron Microscopy

HepG2 cells were seeded in 100-mm cell culture dishes at a density of 100,000 cells/mL and allowed to attach until reaching 90% confluency. Following treatment with OA/PA and ECH for 24 h, cells were collected, washed with PBS, and fixed in a solution containing 2% paraformaldehyde and 2.5% glutaraldehyde in 0.1 M sodium cacodylate buffer for 2–4 h at 4 °C. After washing in 0.1 M sodium cacodylate buffer, cells were postfix-fixed in 2% osmium tetroxide in 0.1 M sodium cacodylate buffer for 2 h. Subsequently, cells were dehydrated through a graded series of alcohols embedded in Epon 816 (Electron Microscopy Sciences, Hatfield, PA, USA), and ultrathin sections (60–80 nm) were prepared using a Leica ultramicrotome (Leica Microsystems, Durham, NC, USA). The prepared sections were then double-stained with uranyl acetate and lead citrate. Subcellular structural changes in the HepG2 cells were ultimately visualized using a transmission electron microscope (TEM, HT7800, Hitachi, Tokyo, Japan).

### 2.6. Immunofluorescence

HepG2 cells were seeded in 24-well plates. Immunofluorescence staining for Nrf2 and GPX4 was performed at a density of 50,000 cells per well. When the cells reached 50% fusion, they were treated with 10 ng/mL Nrf2 and GPX4 binding solution. The cells were then fixed with 4% paraformaldehyde for 15 min and then rinsed with PBS three times. Cells were permeabilized in 3% TritonX-100 for 10 min and blocked in 5% BSA for 1 min. This was followed by incubation with the primary antibodies GPX4 and Nrf2 overnight at 4 °C. After washing with PBS, cells were incubated with Cy3-conjugated secondary antibody for 1 h at room temperature in the dark. Finally, the cells were stained with DAPI for 10 min and observed under a fluorescence microscope (Evos FL Auto, Waltham, MA, USA).

### 2.7. Intracellular Iron Assay

Intracellular iron levels were determined using an iron colourimetric analysis kit (E1042, Applied Original Technology, Beijing, China). 100,000 cells/well were inoculated in 6-well plates. After achieving 80% congruence, cells were lysed with 200 μL lysis buffer per well and placed on a shaker for 2 h. Standards were diluted in equal proportions to create a zero-concentration control reaction group. The buffer was then mixed with a 4.5% solution in a 1:1 ratio, referred to as mixed solution A. Blank, standard, and sample tubes were prepared, mixed well, and incubated at 60 °C for 1 h. After cooling to room temperature, droplets were centrifuged from the cap and transferred to the bottom of the tube. Then 30 μL of ferric ion detection reagent was added, mixed thoroughly, and incubated at room temperature for 30 min. Then 200 µL of the mixture was transferred to a 96-well plate, and the absorbance was measured at 550 nm. A standard curve was plotted, and the concentration of iron ions was calculated.

### 2.8. Glutathione Determination

HepG2 cells were collected, washed twice with PBS, followed by 1 mL RIPA ultrasound, centrifuged for 10 min, and the supernatant was collected at 4 °C for detection. A microreduced glutathione (GSH) assay kit was used for the experiment. The microplate reader (Bio-Rad, Hercules, CA, USA) was warmed up for 30 min, the wavelength was set at 412 nm, and distilled water was adjusted to zero. The reagents were then added and measured using an enzyme marker. Finally, the data obtained were recorded.

### 2.9. Malondialdehyde Determination

HepG2 cells were collected in centrifuge tubes, centrifuged, and the supernatant was discarded. Add 1 mL of the extract for every 5,000,000 cells, and the cells were disrupted by ultrasound. The mixture was then centrifuged at 4 °C, 8000× *g* for 10 min, and the supernatant was transferred to ice for assay. The zymograph was preheated for 30 min and zeroed with distilled water. Reagents were added, and values were measured at 532 nm. Finally, the data were recorded.

### 2.10. Grouping and Administration of Experimental Mice

Experimental mice (SPF grade, 10-week-old, c57BLKS/J db/db, db/m, male) were provided by Changzhou Cavins Laboratory Animal Co., Ltd. (Changzhou, China) and were housed in separate cages of 4–6 mice per cage at the Animal Centre of the Renmin Hospital of Wuhan University (Wuhan, China). The room temperature was 18–24 °C, the relative humidity was 60%, and the light time was 12 h/24 h. The animals were kept at the same temperature and humidity. During the experimental period, the animals were allowed to take food and water freely and were fed with normal feed. The experimental protocol complied with the ethical requirements of the Renmin Hospital of Wuhan University (Wuhan, China) (Issue number: WDRM20190517).

After 2 weeks of acclimatized satisation, db/m mice were assigned to a normal control group (db/m group, n = 6). db/db mice were randomly divided into a MASLD model group (db/db group, n = 6) and an ECH group (db/db + ECH group, n = 6). db/m and db/db groups were supplied with saline (0.05 mL/10 g/day) via intragastric administration, which lasted for 14 weeks. The ECH group was administered ECH (300 mg/kg/day) via intragastric administration. Throughout the 14-week experimental period, the mice had unrestricted access to food and water.

### 2.11. Sample Collection

After 14 weeks of administration, fasting blood glucose (FBG) levels were measured using blood glucose test strips, and blood was collected from the inner corner of the eyes of mice under anaesthesia while the liver was isolated after cardiac perfusion. Liver tissues were preserved, and some of the fresh tissues were cryosectioned and stained with oil red O stain. Some of the tissues were fixed with 4% paraformaldehyde and embedded in paraffin for Oil red, hematoxylin-eosin (HE), and Masson pathology staining, and the rest of the tissues were stored in a −80 °C refrigerator.

### 2.12. Biochemical Indexes of Serum

ALT, AST, TC, TG, high-density lipoprotein cholesterol (HDL-C), and low-density lipoprotein cholesterol (LDL-C) were detected by a biochemical analyzer (ADVID2400, Tokyo, Japan) in the Department of Clinical Laboratory of the Renmin Hospital of Wuhan University (Wuhan, China).

### 2.13. Western Blot

Mice liver tissues were collected, and tissue proteins were extracted. The lysate was assayed by a bicinchoninic acid assay (BCA) to determine the protein concentration. Based on the protein concentration determined by BCA, the total amount of protein in each group of samples was standardised to 20 μg. Protein samples were separated on 8–12% SDS polyacrylamide gels and transferred to PVDF membranes using the Bio-Rad system. The membranes were blocked with 5% BSA for 1 h at room temperature and then incubated with primary antibodies against Nrf2 (1:1000, wanleibio, Wuhan, China), HMOX-1 (1:1000, wanleibio, Wuhan, China), GPX4 (1:1000, wanleibio, Wuhan, China), SLC7A11 (1:1000, Proteintech, Wuhan, China), and ACSL4 (1:1000, ABclonal, Wuhan, China) overnight at 4 °C. After washing with TBST, the membranes were incubated with the corresponding secondary antibodies (1:5000, Proteintech, Wuhan, China) for 1 h at room temperature. Finally, the membranes were developed using an enhanced ECL kit (Thermo Fisher Scientific, Waltham, MA, USA). The relative protein expression compared with the internal control was calculated using ImageJ (1.54f, National Institutes of Health, Bethesda, MA, USA).

### 2.14. Statistical Analysis

All in vitro experiments were repeated at least three times. Values are expressed as mean ± standard deviation (SD) standard error (SEM). Data were analyzed using one-way analysis of variance (ANOVA) followed by Tukey’s post hoc test. *p*-values less than 0.05 were considered statistically significant.

## 3. Results

### 3.1. Effects of ECH on Cell Viability

**Figure 1:**
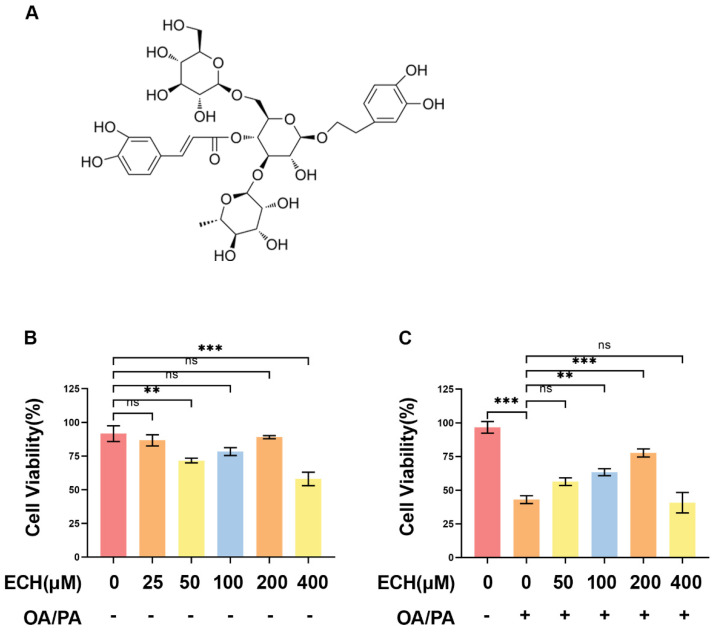
Effect of ECH on cell viability. (**A**) Molecular structure of echinacoside (ECH). (**B**) The effects of different concentrations of ECH (0, 25, 50, 100, 200, 400 μM) on HepG2 cell viability. (**C**) The effect of ECH (0, 50, 100, 200, 400 μM) on the viability of HepG2 cells with or without OA/PA (150/75 μM/mL). Significance: ns, *p* ≥ 0.05; **, *p* < 0.01; ***, *p* < 0.001.

The chemical structure of ECH is illustrated in Figure 1A. As shown in Figure 1B, there was no notable difference in cell viability among the 25, 50, 100, and 200 μM groups; ECH concentrations ranging from 100 to 200 μM did not exhibit significant toxic effects on the cells. Exposure to OA/PA (150/75 μM/mL) resulted in decreased HepG2 cell viability compared to the control group, whereas ECH at a concentration of 200 μM significantly reversed the reduction in cell viability in OA/PA-treated cells (Figure 1C). Consequently, we selected 200 μM ECH for subsequent experiments.

### 3.2. Fer-1 and ECH Alleviated OA/PA-Induced Lipid Deposition and Lipid Peroxidation

**Figure 2:**
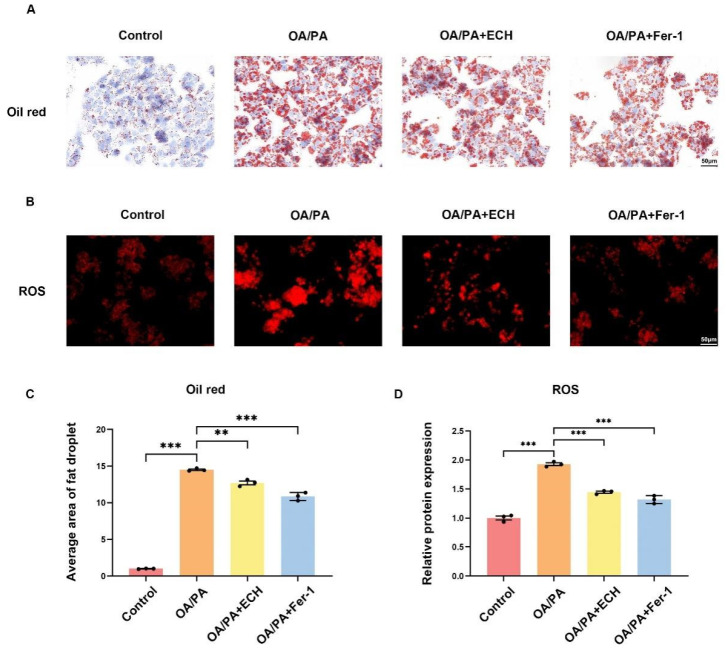
Fer-1 and ECH alleviate lipid deposition and lipid peroxidation caused by OA/PA. (**A**) Oil red O staining (400×, scale = 50 μm). (**B**) The immunofluorescence staining of ROS (400×, scale = 50 μm). The red fluorescence intensity reflects the protein expression level of ROS. (**C**) Measurement of the average area of fat droplets. (**D**) ROS fluorescence scale. Significance: ns, *p* ≥ 0.05; **, *p* < 0.01; ***, *p* < 0.001.

As can be seen from (Figure 2A,C), intracellular lipid droplets were significantly increased in OA/PA-treated HepG2 cells and significantly decreased after ECH treatment. This proved that severe lipid deposition occurred in OA/PA-treated HepG2 cells, and the addition of ECH could inhibit lipid deposition to some extent. Meanwhile, as shown in (Figure 2B,D), the red fluorescence of cellular lipid oxidation product ROS was enhanced in OA/PA-treated HepG2 cells, while Fer-1 and ECH treatment could also reduce the fluorescence intensity of cellular ROS. In summary, the results confirmed that ECH could alleviate OA/PA-induced lipid and lipid peroxidation product deposition in HepG2 cells, which could further delay the development of MASLD.

### 3.3. Fer-1 and ECH Maintained the Dissipation of the Mitochondrial Membrane Potential in Ferroptosis

**Figure 3:**
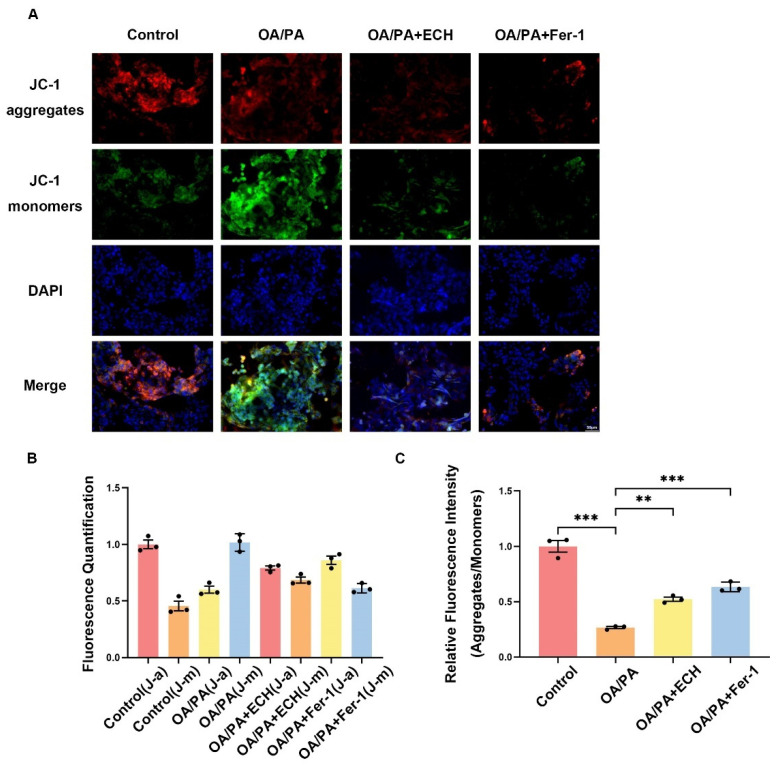
Effects of Fer-1 and ECH on mitochondria of HepG2 cells under OA/PA. (**A**) Monitoring of mitochondrial membrane potential by JC-1 staining (400×, scale = 50 μm). The ratio of red/green fluorescence indicates the degree of mitochondrial damage. (**B**) Fluorometric scale of JC-1 (J-a indicates JC-1aggregates, J-m indicates JC-1monomers). (**C**) Relative fluorescence intensity (aggregates/monomers) of JC-1. Significance: **, *p* < 0.01; ***, *p* < 0.001.

Abnormal changes in mitochondrial membrane potential are indicative of mitochondrial dysfunction and have been identified as a marker of ferroptosis in recent years. To clearly investigate the effects of Fer-1 and ECH on the mitochondrial membrane potential during ferroptosis in HepG2 cells, we conducted JC-1 staining. As can be seen in Figure 3A,B, after exposure of HepG2 cells to OA/PA, a large number of JC-1 aggregates (reflected in red fluorescence intensity) were separated into JC-1 monomers (reflected in green fluorescence intensity). The ratio of red/green fluorescence intensity decreased significantly, indicating an increased degree of mitochondrial damage. Interestingly, the decrease or loss of mitochondrial membrane potential was partially restored after the use of ECH. ECH counteracted the OA/PA-induced alteration of mitochondrial membrane potential and partially restored mitochondrial function. It is demonstrated that ECH plays a role in protecting mitochondrial membrane potential from damage during OA/PA-induced ferroptosis.

### 3.4. Fer-1 and ECH Abolished the Elevation Impairments of the Mitochondrial Ultra-Structures

**Figure 4:**
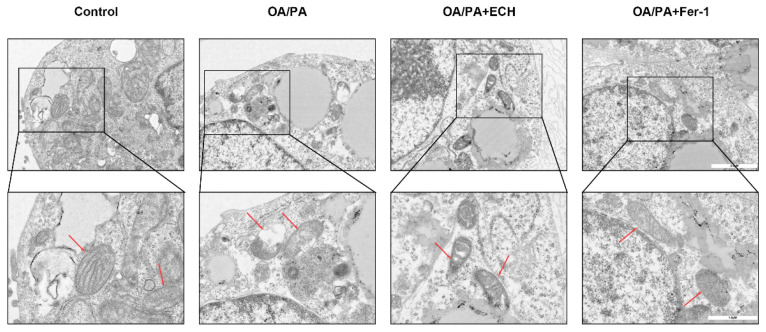
Mitochondrial changes in HepG2 cells treated with the indicated concentrations of Fer-1 and ECH. The ultrastructures of mitochondria were observed by transmission electron microscopy (the first row: 7.0k×, scale = 2 μm; the second row: 15.0k×, scale = 1 μm). Mitochondrial shrinkage, reduced or disappeared mitochondrial cristae, and ruptured outer mitochondrial membrane were observed in OA/PA-treated HepG2 cells. Red arrows point to mitochondria.

**Figure 5:**
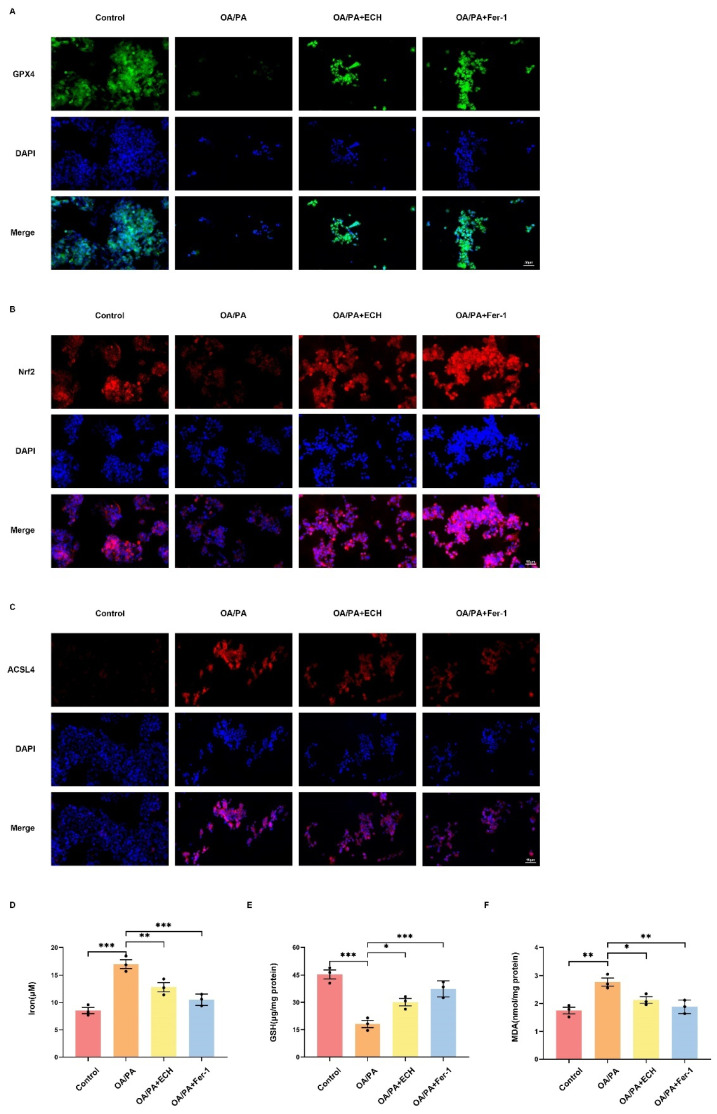
Fer-1 and ECH inhibits OA/ PA-induced ferroptosis by affecting the expression of signaling pathway proteins in HepG2 cells. (**A**) The total expression level of GPX4 in HepG2 cells was detected by immunofluorescence staining (400×, scale = 50 μm). The green fluorescence intensity reflected the expression level of GPX4 protein. The blue fluorescence reflects nuclear (DAPI is a DNA-specific probe). (**B**) The total expression level of Nrf2 in HepG2 cells was detected by immunofluorescence staining (400×, scale = 50 μm). The expression level of Nrf2 protein was reflected by red fluorescence intensity. The blue fluorescence reflects nuclear. (**C**) The total expression level of ACSL4 in HepG2 cells was detected by immunofluorescence staining (400×, scale = 50 μm). The red fluorescence intensity reflected the expression level of ACSL4 protein. The blue fluorescence reflects nuclear. (**D**–**F**) The contents of iron, GSH, and MDA in the cells were determined by the corresponding detection kit. Significance: *, *p* < 0.05; **, *p* < 0.01; ***, *p* < 0.001.

To further investigate the altered mitochondrial morphology, we utilized transmission electron microscopy to evaluate the ultrastructure of mitochondria. As previously reported, characteristic changes of ferroptosis, including mitochondrial contraction and deformation, rupture of the outer mitochondrial membrane, and reduction or disappearance of mitochondrial cristae, were observed in OA/PA-treated HepG2 cells. However, deleterious changes to mitochondrial morphology were inhibited by the use of ECH and Fer-1 (Figure 4). Iron overload is the hallmark and basis of ferroptosis, which triggers a series of subsequent adverse effects, including ROS accumulation. As anticipated, ECH suppressed the increase in intracellular iron level induced by OA/PA (Figure 5D). Intracellular iron is imported into the mitochondria, and excess iron in the mitochondria mediates the massive production of ROS via the Fenton reaction, ultimately leading to cellular ferroptosis. We can conclude that ECH reduced intracellular iron content while significantly reversing OA/PA-stimulated ferroptosis in HepG2 cells. This suggests that ferroptosis actually occurs in the cellular model of MASLD and that ECH plays a protective role in the progression of MASLD by inhibiting cellular ferroptosis.

### 3.5. Fer-1 and ECH Ameliorates Ferroptosis in HepG2 Cells by Regulating the Nrf2/HMOX-1/SLC7A11/GPX4 Pathway

OA/PA is a typical common inducer of MASLD cell modeling, and studies have confirmed that it also triggers cellular ferroptosis [19]. Iron overload is the hallmark and basis of ferroptosis, where intracellular iron enters the mitochondria, and excess iron in the mitochondria mediates the massive production of ROS via the Fenton reaction, ultimately leading to cellular ferroptosis. As expected, OA/PA increased intracellular iron levels, and Fer-1 and ECH inhibited the OA/PA-induced increase in intracellular iron (Figure 5D), reversing OA/PA-stimulated ferroptosis in HepG2 cells. GSH, an essential intracellular antioxidant system, is indispensable for the function of GPX4, and its depletion is a characteristic change observed during the development of ferroptosis. The results showed that GSH depletion was enhanced in the OA/PA group compared with the control group, whereas Fer-1 and ECH increased intracellular GSH content and partially reduced depletion (Figure 5E). In addition, OA/PA resulted in the overproduction of intracellular MDA, a further lipid peroxidation metabolite. Fer-1 and ECH administration reduced the enhanced intracellular MDA levels (Figure 5F).

**Figure 6:**
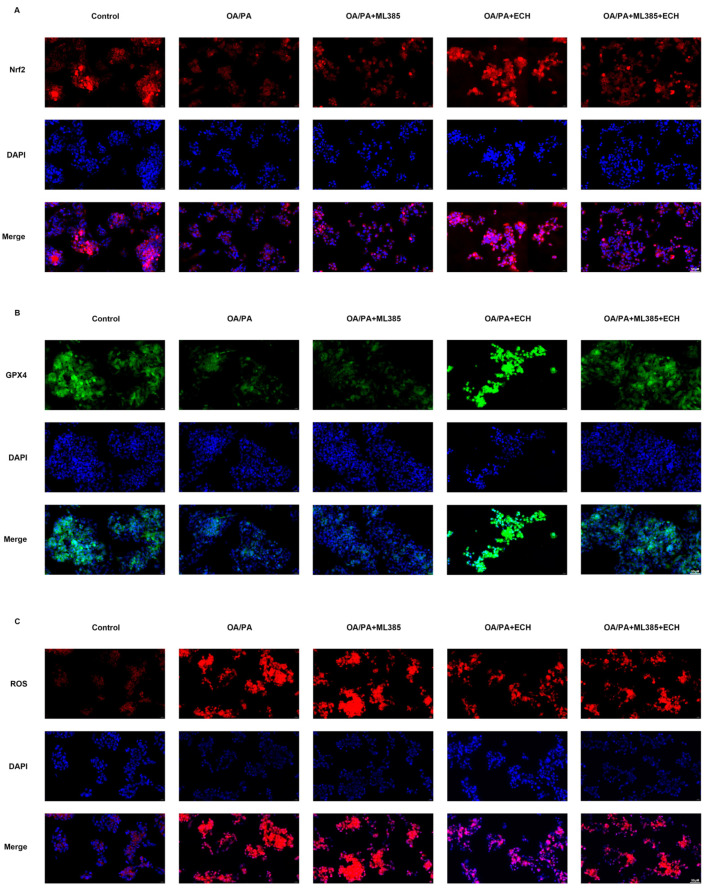
ML385 impacts the inhibitory effect of ECH on ferroptosis in HepG2 cells. (**A**) The total expression level of Nrf2 in HepG2 cells was detected by immunofluorescence staining (400×, scale = 50 μm). The expression level of Nrf2 protein was reflected by red fluorescence intensity. The blue fluorescence reflects nuclear. (**B**) The total expression level of GPX4 in HepG2 cells was detected by immunofluorescence staining (400×, scale = 50 μm). The green fluorescence intensity reflected the expression level of GPX4 protein. The blue fluorescence reflects nuclear. (**C**) The total expression level of ROS in HepG2 cells was detected by immunofluorescence staining (400×, scale = 50 μm). The red fluorescence intensity reflected the expression level of ROS. The blue fluorescence reflects nuclear.

In cellular immunofluorescence staining, OA/PA led to a decrease in the intensity of GPX4 green fluorescence and Nrf2 red fluorescence visible in the cytoplasm and an increase in the intensity of ACSL4 red fluorescence. This was reversed by Fer-1 and ECH (Figure 5A–C). The above reversal of ECH was attenuated after the use of an Nrf2 inhibitor (ML385), demonstrating that the drug can exert a protective effect by modulating Nrf2 (Figure 6A–C). The aforementioned phenomena are representative of the events that occur during iron metamorphosis. These data collectively indicate that ECH may act to inhibit iron metamorphosis via the Nrf2/HMOX-1 pathway, thereby attenuating the damage observed in OA/PA-treated HepG2 cells.

### 3.6. ECH Attenuates Lipid Metabolism Disorders and Liver Function Impairment in db/db Mice

**Figure 7:**
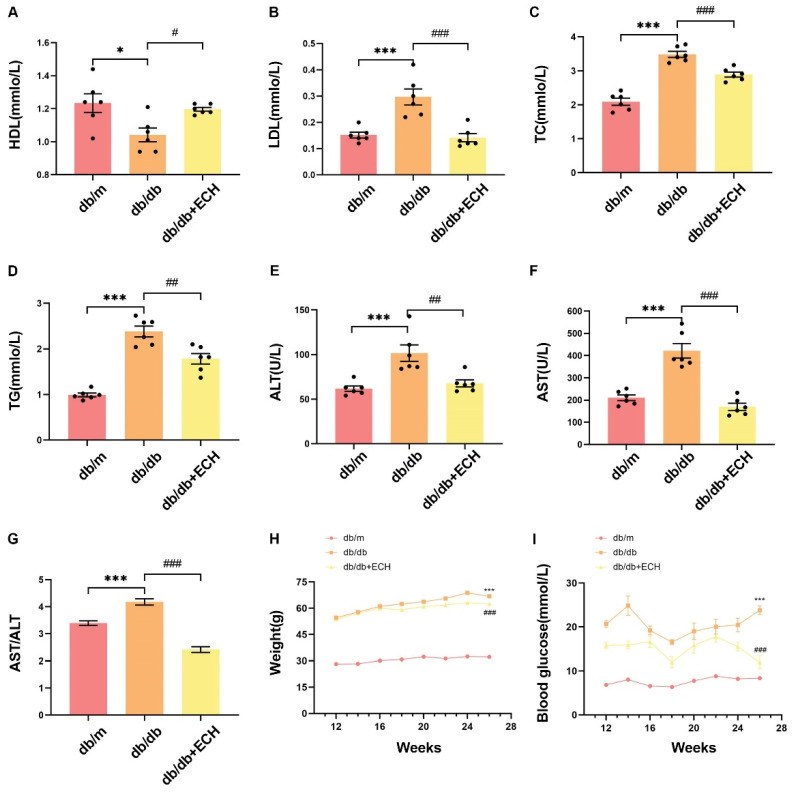
ECH alleviated lipid metabolism disorders and impaired liver function in mouse models. (**A**–**D**) Serum HDL-C, LDL-C, TC, TG levels. (**E**,**F**) Serum ALT, AST levels. (**G**) Serum AST/ALT level. (**H**) Weight gain; circle = db/m group, rectangle = db/db group, triangle = db/db + ECH group. (**I**) FPG; circle = db/m group, rectangle = db/db group, triangle = db/db-ECH group. Significance: *, *p* < 0.05; ***, *p* < 0.001 vs. db/m; #, *p* < 0.05; ##, *p* < 0.01; ###, *p* < 0.001 vs. db/db.

To further investigate the inhibitory effect of ECH on the pathologic process of MASLD, we used mice for in vivo studies. As Figure 7A–D, we found that TC, TG, and LDL-C levels were elevated in the db/db group compared with the db/m group, while HDL-C levels were decreased, suggesting that hyperlipidemia and impaired lipid metabolism occurred in MASLD model mice. Compared with the db/db group, TC, TG, and LDL-C were decreased while HDL-C was increased in the ECH group. When hepatocytes are damaged or necrotic, ALT, AST, γ-glutamyl transpeptidase, and alkaline phosphatase are released from the cells into the serum, and thus the levels of these enzymes reflect the extent and type of liver injury. Elevated ALT and AST are associated with increased mortality from liver disease [20]. As shown in Figure 7E–G, ALT, AST, and AST/ALT levels in the db/db group were higher than those in the db/m group, suggesting that liver dysfunction existed in the model mice. The ALT, AST and AST/ALT levels in the ECH group were lower than those in the db/db group, and the liver dysfunction was somewhat alleviated. Changes in body weight and fasting plasma glucose (FPG) were examined fortnightly in all three groups. The db/db + ECH and db/db groups had higher body weight and weight gain than the db/m group (Figure 7H). However, weight gain was relatively low in the db/db + ECH group compared to the db/db group. Similarly, FPG levels were significantly higher and fluctuated sharply in db/db mice compared to the db/m group (Figure 7I), and both FPG levels and fluctuations decreased after the ECH intervention compared to the db/db group. We can conclude that ECH can reduce the body weight and FPG level of db/db mice, and improve the lipid metabolism disorder and liver dysfunction of db/db mice to some extent.

### 3.7. ECH Attenuates the Progression of MASLD in db/db Mice

**Figure 8:**
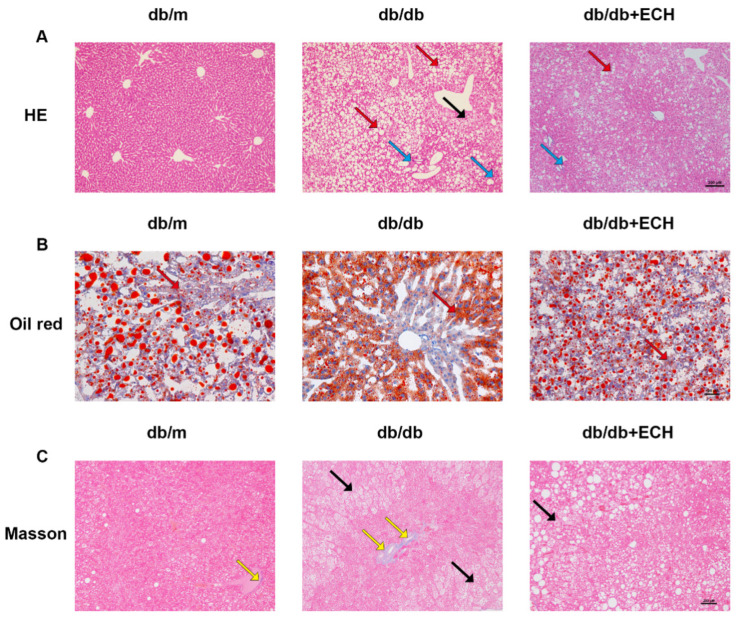
Effects of ECH on liver morphology, lipid accumulation and collagen deposition in liver tissue. (**A**) HE staining of liver tissue (200×, scale = 50 μm). (**B**) Oil red O staining of liver tissue (200×, scale = 50 μm). (**C**) Masson staining of liver tissue (200×, scale = 50 μm). The red arrows pointing to the lipid droplet, the blue arrows pointing to inflammatory cells; the black arrows pointing to balloon-like degenerated cells; the yellow arrows pointing to fibrosis in perisinusoidal tissues.

As, (Figure 8A), HE staining of liver tissue showed normal size and morphology of hepatocytes with clear lobules and sinusoids in the db/m group. The db/db group had extensive degeneration of hepatocytes with ballooning changes, increased hepatocyte volume, and infiltration of inflammatory cells in the portal area, whereas the ECH group had a reduced number of balloon-like degenerated cells and inflammatory cells. As shown in (Figure 8B) oil red O staining of liver tissue, a small number of lipid droplets were present in the cytoplasm of hepatocytes in the db/m group. Several fat particles of different sizes were seen in the hepatocytes of the db/db group, while fat droplets were significantly reduced in the ECH group. We also observed the distribution of collagen in the liver tissue (stained blue) by Masson staining (Figure 8C). Hepatic lobules in the db/m group had a clear and intact structure, and there was no hepatic fibrosis. In the db/db group, the normal structure of the hepatic lobules was isrupted and the hepatic cords were disorganized. In addition, obvious fibrous tissue proliferation was observed. Collagenous tissue proliferation was reduced in the ECH-treated group. The above results suggested that ECH reduced histopathological damage and lipid deposition in the liver to a certain extent and also reduced hepatic fibrosis and delayed the disease process, which was consistent with the in vitro experiments.

### 3.8. ECH Ameliorates Ferroptosis in Liver Tissue by Regulating the Nrf2/HMOX-1/SLC7A11/GPX4 Pathway

**Figure 9:**
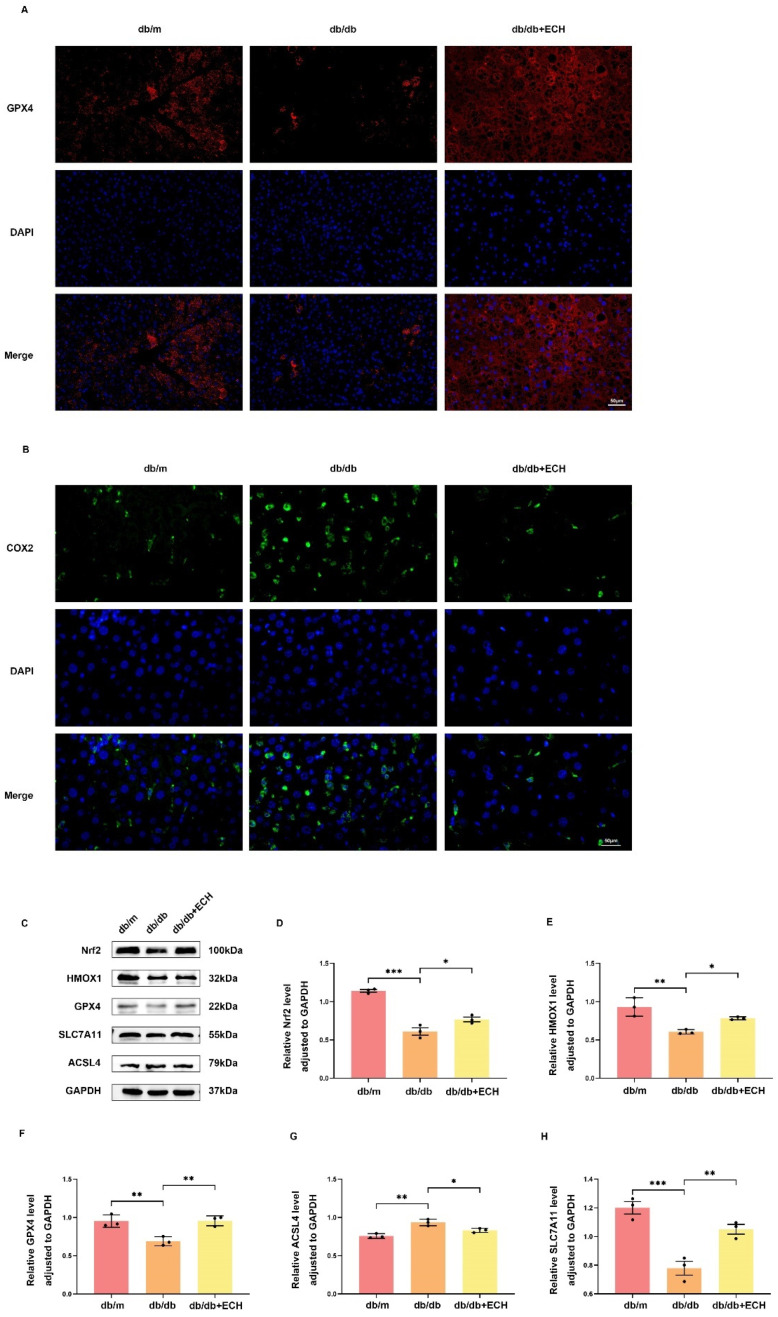
ECH inhibits ferroptosis by regulating the Nrf2/HMOX-1/SLC7A11/GPX4 signaling pathway in mouse liver tissue. (**A**) The total expression level of GPX4 in mouse liver tissues was detected by immunofluorescence staining (400×, scale = 50 μm). Red fluorescence intensity reflects GPX4 protein expression level. The blue fluorescence reflects nuclear. (**B**) The total expression level of COX2 in mouse liver tissues was detected by immunofluorescence staining (400×, scale = 50 μm). Red fluorescence intensity reflected the expression level of COX2 protein. The blue fluorescence reflects nuclear. (**C**) Western blot detected the protein expression levels of Nrf2, HMOX-1, SLC7A11, GPX4, and ACSL4. (**D**–**H**) Relative protein expression was measured by densitometry. GAPDH was used as internal control. Significance: *, *p* < 0.05; **, *p* < 0.01; ***, *p* < 0.001.

SLC7A11, GPX4, and ACSL4 are widely recognized as biomarkers of ferroptosis and are considered downstream regulators of this process [21]. Nrf2 is among the many upstream regulatory genes that act on SLC7A11 [11]. Nrf2/HMOX-1/SLC7A11/GPX4 is a classical pathway for ferroptosis [22,23]. The results of liver tissue protein blot showed that ECH reversed the decreased expression of Nrf2, HMOX-1, SLC7A11, and GPX4 and increased expression of ACSL4 in MASLD to some extent (Figure 9C–H).

In addition, in immunofluorescence staining of liver tissues, the db/db group showed diminished intensity of GPX4 red fluorescence and enhanced COX2 (encoded by the PTGS2 gene) green fluorescence compared with the db/m group, whereas the ECH group reversed the above changes in db/db mice (Figure 9A,B). All of the above phenomena are representative events in the progression of ferroptosis. Taken together, these data demonstrated that ECH could inhibit ferroptosis via the Nrf2/HMOX-1/SLC7A11/GPX4 pathway, thereby attenuating the liver injury in db/db mice.

## 4. Discussion

Metabolic dysfunction-associated steatotic liver disease (MASLD) is a group of liver diseases associated with the metabolic syndrome that affects approximately 1.7 billion people worldwide. The severity of the disease can progress from hepatic steatosis to metabolic dysfunction-associated steatohepatitis (MASH) and can continue to advanced liver fibrosis, cirrhosis, and even hepatocellular carcinoma (HCC). A significant proportion of MASLD patients also suffer from metabolic syndrome, which increases the risk of cardiovascular disease and extrahepatic cancers in MASLD patients [24]. MASLD has become the most common liver disease in China, with more than 240 million patients. The prevalence of MASLD has reached 27% in the urban population, which will lead to a significant increase in the burden of MASLD-associated advanced liver disease and the overall disease burden [25]. However, we currently have no systematic and effective therapeutic strategies for MASLD, so it is important to explore drugs with novel mechanisms of action for the prevention and treatment of MASLD. 

Cistanche tubiflora is a traditional plant with hepatoprotective, nephroprotective, and intestinal properties. Echinacoside (ECH), an active constituent of Cistanche tubiflora, has multiple biological effects, including anti-oxidative stress and anti-inflammatory effects, and shows hepatoprotective effects by ameliorating oxidative stress, inflammation, and cell death [26]. This study is the first to demonstrate that ECH delays the development of MASLD by inhibiting ferroptosis and protecting mitochondrial function in hepatocytes.

In addition to apoptosis, autophagy, senescence, and necrosis, several reports have indicated that hepatic iron concentration may also play a role in initiating inflammation in MASLD [27]. OA/PA has been identified as a typical inducer in the in vitro MASLD models. The results demonstrated that treatment of HepG2 cells with OA/PA at concentrations of 150/75 μM/mL resulted in a reduction in cell viability, an increase in lipid deposition, an elevation in the level of a representative lipid peroxidation product, ROS, and the formation of lipid deposits and ferroptosis in the cells. The aforementioned indicators were to some extent alleviated following the application of ECH. It is noteworthy that COX2 has been identified as a key enzyme in prostaglandin synthesis, which represents a critical step in the generation of lipid ROS. Previous studies have demonstrated that upregulation of PTGS2 (encoding COX2) could serve as a suitable biomarker for GPX4-regulated ferroptosis [28]. The results demonstrated that in the livers of db/db mice, hepatic PTGS2 expression was elevated, hepatocytes exhibited extensive degeneration with globular changes, hepatocyte volume was increased, several different sizes of fat particles were observed in hepatocytes, there were inflammatory cells infiltrating in the portal area, the normal structure of the hepatic lobules was damaged, and the hepatic cords were disorganized, with the pathological manifestations of MASLD. db/db mice are leptin receptor-deficient. Leptin, an adipose-derived cytokine that prevents the invasion and accumulation of free fatty acids in non-adipose tissues, is an anti-lipotropic hormone [29]. When leptin receptor-deficient, the body loses protection against lipotoxicity, steroid regulatory element binding protein gene expression is upregulated, free fatty acids accumulate in the liver, and hepatocellular steatosis occurs. Serum leptin has a pro-inflammatory effect, and hyperleptinemia promotes the development of hepatic fibrosis, leading to the development of MASLD associated with lipotoxicity. It may co-occur in parallel or interact with the lipid peroxidation and further ferroptosis playing an important role in the development of MASLD. The serum levels of alanine aminotransferase (ALT), aspartate aminotransferase (AST), triglycerides (TG), total cholesterol (TC) and low-density lipoprotein (LDL) were elevated, and serum changes could also be used as an indicator of the degree of hepatic lipid accumulation [30]. The administration of ECH resulted in a reduction in hepatic PTGS2 expression, hepatic steatosis, lipid droplet deposition and the number of dead hepatocytes in MASLD mice. Furthermore, ECH was observed to significantly reduce serum ALT, AST, TG, TC, and LDL levels. The results of this study indicate that ECH may influence fatty acid metabolism, lipid accumulation, ferroptosis and liver damage, and may contribute to the alleviation of disease progression in MASLD.

It would appear that there is currently no established guideline for the assessment of ferroptosis in existing HepG2 models. In the present study, the most widely recognised indicators were therefore tested. Ferroptosis is essentially the result of an imbalance in the oxidative/antioxidant system. The most abundant intracellular antioxidant, GSH, acts as a cofactor for GPX4, whose synthesis is influenced by the xc-mediated cystine transport system. GPX4 is a key regulator of ferroptosis, an antioxidant enzyme, which catalyses the reduction of lipid peroxides in the presence of GSH [31]. In terms of the underlying mechanisms, SLC7A11, GPX4, GSH, and MDA (the end product of lipid peroxidation), iron levels, ROS and COX2 can be attributed to molecular events associated with ferroptosis. In the present study, it was observed that in OA/PA-treated HepG2 cells, there was an elevation in intracellular iron, ROS, MDA, and ACSL4 levels, while Nrf2, GPX4, and GSH levels were found to be downregulated. These findings indicate that ferroptosis occurs in the MASLD model in-vitro. Following the administration of ECH, the aforementioned indicators exhibited varying degrees of alleviation, thereby indicating that ECH could potentially mitigate OA/PA-induced ferroptosis in HepG2 cells.

The evidence indicates that the SLC7A11-GSH-GPX4 axis represents a significant cellular system that protects against ferroptosis [32]. The transcription factor Nrf2 (nuclear factor erythroid 2-related factor 2) activates endogenous antioxidant response elements and plays a role in the transcriptional regulation of numerous genes related to ferroptosis. The Nrf2 transcription factor plays a pivotal role in the attenuation of lipid peroxidation and iron concentration [18] and further promotes the transcription of downstream antioxidant enzyme genes (e.g., HMOX-1 and GPX4) to exert antioxidant capacity [33]. HMOX-1 plays a role in the metabolism process and is an important antioxidant enzyme. On the one hand, the degradation of the heme moiety facilitates the blocking of its pro-oxidant effect. Conversely, the by-product biliverdin and its reduced form, bilirubin, have been demonstrated to exhibit antioxidant activity by scavenging ROS. It is therefore evident that these factors play a pivotal role in the regulation of ferroptosis, exerting influence through the modulation of the GSH antioxidant system and iron metabolism. Furthermore, the present study demonstrated that ECH significantly up-regulated the levels of Nrf2, HMOX-1, GPX4, and SLC7A11 and down-regulated the expression level of ACSL4 in the liver tissues of MASLD model mice. The Nrf2/HMOX-1/SLC7A11/GPX4 pathway may be a pivotal regulator of ferroptosis in MASLD.

**Figure 10:**
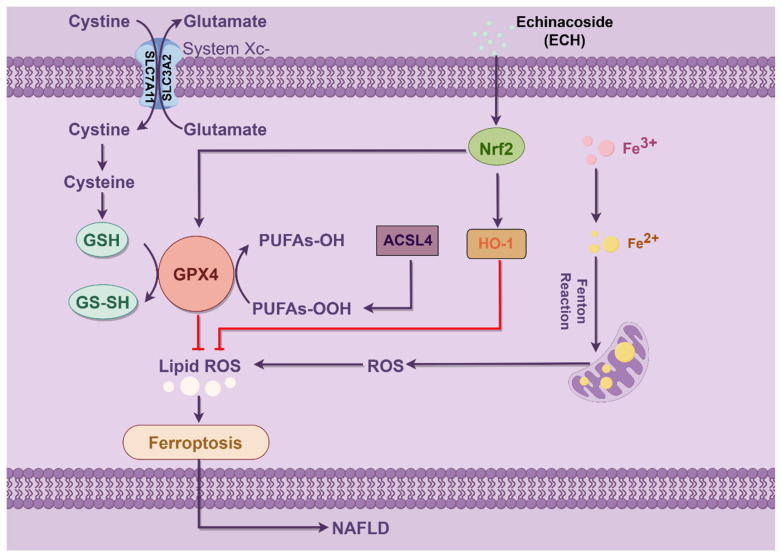
The effect of echinacoside in HepG2 cells. ECH was observed to upregulate the levels of Nrf2 and its downstream HMOX-1, GPX4, and SLC7A11 while inhibiting the expression of ACSL4 in a MASLD cell model. The imbalance of redox reactions in MASLD was regulated through the Nrf2/HMOX-1/SLC7A11/GPX4 pathway, and the massive release of intracellular ROS was inhibited, which protected the mitochondria, inhibited lipid peroxidation of the cell membrane structure, and ultimately inhibited the occurrence of ferroptosis. ECH alleviates MASLD by inhibiting ferroptosis via the Nrf2/HMOX-1 pathway. The schematic diagram was drawn by figdraw.

Ferroptosis is defined by the occurrence of membrane lipid peroxidation, which is induced by the Fenton reaction and is accompanied by a significant release of intracellular ROS (Figure 10). This, in turn, promotes further lipid peroxidation of the cell membrane structure, which ultimately results in the promotion of ferroptosis [34]. Mitochondria possess a bilayer membrane structure and play a pivotal role in the regulation of cell death, encompassing apoptosis, autophagy, and necrosis [35]. It is therefore reasonable to hypothesize that mitochondria are important target subcellular organelles in patients with ferroptosis. From a mechanistic perspective, the involvement of mitochondria in ferroptosis is closely related to the function of classical mitochondrial metabolic activities. This is probably due to the mitochondrial electron transport chain (ETC) and the mitochondrial tricarboxylic acid (TCA) cycle, which favor the production of ROS [36]. In the presence of excess iron, hydrogen peroxide reacts to produce a significant quantity of hydroxyl radicals (OH), which subsequently oxidize polyunsaturated fatty acids (PUFAs) on cell membrane structures to lipid hydroperoxide derivatives (LOOH) [3,37]. Intracellular iron is transported to mitochondria via SLC25A37 and SLC25A28, and thus impaired intracellular and mitochondrial iron homeostasis has been identified as a hallmark of iron overload, which in turn accelerates the process of iron overload [38]. In the present study, we observed elevated iron levels in HepG2 cells subjected to OA/PA-induced ferroptosis, while treatment with ECH resulted in a partial reduction in the observed increase in iron. This indicates that ECH may play a pivotal role in the resistance to ferroptosis in an in vitro model of MASLD. In addition, the TCA cycle and the ETC are involved in ATP production via the membrane potential of the mitochondria, and changes in mitochondrial membrane potential are an early warning signal for ferroptosis [14]. The results demonstrated that HepG2 cells treated with OA/PA exhibited a reduction in mitochondrial membrane potential, while the application of Fer-1 and ECH impeded this decline. The present study demonstrated that ECH mitigated the deleterious effects of HepG2 cell mitochondria on OA/PA-induced ferroptosis. These effects included rupture and vacuolization of the outer membrane, a reduction in mitochondrial volume, and a reduction or absence of mitochondrial cristae. Based on these findings, it can be concluded that ECH exerts a significant protective effect against OA/PA-mediated ferroptosis by attenuating mitochondrial damage.

Nevertheless, it should be noted that this study is not without its own set of limitations. For instance, the specific targets of ECH in vivo remain unclear, and the existence of additional mechanisms of action requires further investigation. Furthermore, MASLD is a complex disease state characterized by hepatic lipid deposition, lipid peroxidation, inflammation, and fibrosis. Therefore, it is not possible to achieve a complete cure by targeting only one mechanism or pathway. It remains unclear whether ECH also exhibits this protective effect in humans, which are more complex than mice. Furthermore, the optimal therapeutic dose and toxic side effects of ECH in mice and humans remain unknown. It can therefore be concluded that there is still a considerable way to go before ECH can be used for clinical treatment.

## 5. Conclusions

In conclusion, this research provides credible evidence for the involvement of ferroptosis in the pathogenesis and progression of MASLD and reports the anti-lipid deposition and ferroptosis inhibiting properties of ECH in MASLD for the first time. In addition, it was demonstrated that ECH slows down the progression of MASLD by regulating mitochondrial function and inhibiting ferroptosis through the Nrf2/SLC7A11/GPX4 signaling pathway. Our study will contribute to the understanding of the therapeutic efficacy of ECH and provide new ideas for the future development of targeted anti-ferroptosis therapy for MASLD.

## Data Availability

Data will be made available on request.
